# The DASH diet in diabetes related complications or comorbidities: an unexpected friend

**DOI:** 10.3389/fnut.2025.1689467

**Published:** 2025-10-28

**Authors:** Kai Liu, Shu Liu, Dong Wang, Hong Qiao

**Affiliations:** ^1^Department of Endocrinology, Second Affiliated Hospital of Harbin Medical University, Harbin, Heilongjiang, China; ^2^Health Management Centre, Fourth Affiliated Hospital of Harbin Medical University, Harbin, Heilongjiang, China; ^3^Scientific Research Centre, Second Affiliated Hospital of Harbin Medical University, Harbin, China

**Keywords:** DASH diet, diabetes, complications, inflammatory factor, oxidative stress

## Abstract

The global epidemic of diabetes and its complications poses a serious challenge to public health. Metabolic disorders and chronic hyperglycemia drive multi-system damage. The application of Dietary Approaches to Stop Hypertension (DASH) diet has been extended from hypertension management to multi-dimensional integrated prevention and treatment of diabetes. DASH diet significantly reduces the risk of type 2 diabetes by optimizing blood glucose homeostasis, reducing hemoglobin A1c (HbA1c), improving insulin sensitivity and insulin resistance, regulating lipid metabolism, and inhibiting oxidative stress and inflammation. In terms of management of complications, the DASH diet reduces the risk of diabetic nephropathy and delays decline of renal function. Its antihypertensive effect and improvement in arterial elasticity synergistically reduce the risk of cardiovascular events. The diet has also shown regulatory potential for metabolic abnormalities in polycystic ovary syndrome and microvascular damage in diabetic retinopathy. The DASH diet is suitable for long-term health management due to its advantages of standardized regimens and multi-target metabolic regulation. Future research needs to focus on molecular mechanisms, individual application optimization, and cross-disease synergies to strengthen the scientific basis and practical value in the comprehensive management of diabetes. This review discusses the multiple abilities by which the DASH diet provides comprehensive protection against diabetes and its complications or comorbidities.

## Graphical abstract

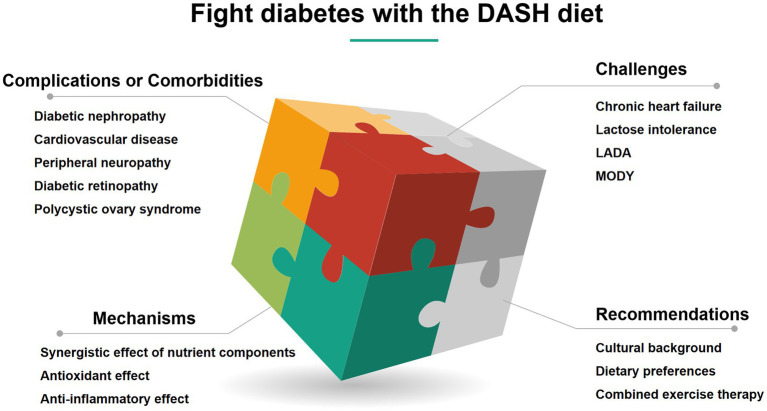

## Highlights

- Multi-target protection against diabetes complications: The DASH diet demonstrates comprehensive benefits in reducing diabetic nephropathy risk, improving cardiovascular health, and mitigating oxidative stress and inflammation, thereby delaying microvascular and macrovascular complications.
- Cross-disease metabolic synergy: Beyond diabetes, the DASH diet shows efficacy in managing gestational diabetes, polycystic ovary syndrome (PCOS), and diabetic retinopathy through its anti-inflammatory, antioxidant, and insulin-sensitizing properties.
- Tailored adaptations for special populations: The diet is adaptable for elderly patients, individuals with chronic heart failure, or lactose intolerance, emphasizing sodium restriction, nutrient-dense alternatives, and cultural customization to ensure safety and adherence.
- Future directions for precision nutrition: Research gaps include elucidating molecular mechanisms, optimizing individualized dietary strategies, and exploring cross-disease synergies to enhance the DASH diet’s role in holistic diabetes management.

## 1 Introduction

Diabetes is one of the most common chronic diseases in the world, and its complications place a heavy burden on individual health and the social economy. Diabetes mellitus is characterized by chronic hyperglycemia, which leads to metabolic disorders in multiple systems of the body and then to a variety of microvascular and macrovascular complications (1, 2). According to statistics, metabolic diseases have affected about a quarter of the world’s population, and their incidence has been rising rapidly in the past few decades. Type 2 diabetes mellitus (T2DM) is dominant, and is closely related to obesity and metabolic syndrome (2, 3). About 15% of people in developed countries have a combination of diabetes, obesity and high blood pressure (“diabesotension “), which doubles the risk of cardiovascular disease (3). Complications of diabetes include microvascular disease (e.g., retinopathy, nephropathy, neuropathy) and macrovascular disease (e.g., coronary heart disease, stroke). Insulin resistance interferes with adipose tissue remodeling, pancreatic β-cell function and energy metabolism regulation, leading to a vicious cycle of glucose and lipid metabolism disorders, and eventually leading to diabetic macrovascular complications (4, 5). The oxidative stress and inflammation caused by chronic hyperglycemia can lead to diabetic neuropathy (DN), retinopathy and other complications. Diabetic retinopathy (DR) is one of the most common microvascular complications of diabetes, with a high incidence leading to visual impairment and even blindness (1). In addition, cardiovascular disease is the main cause of death in patients with diabetes (6, 7).

**Figure 1:**
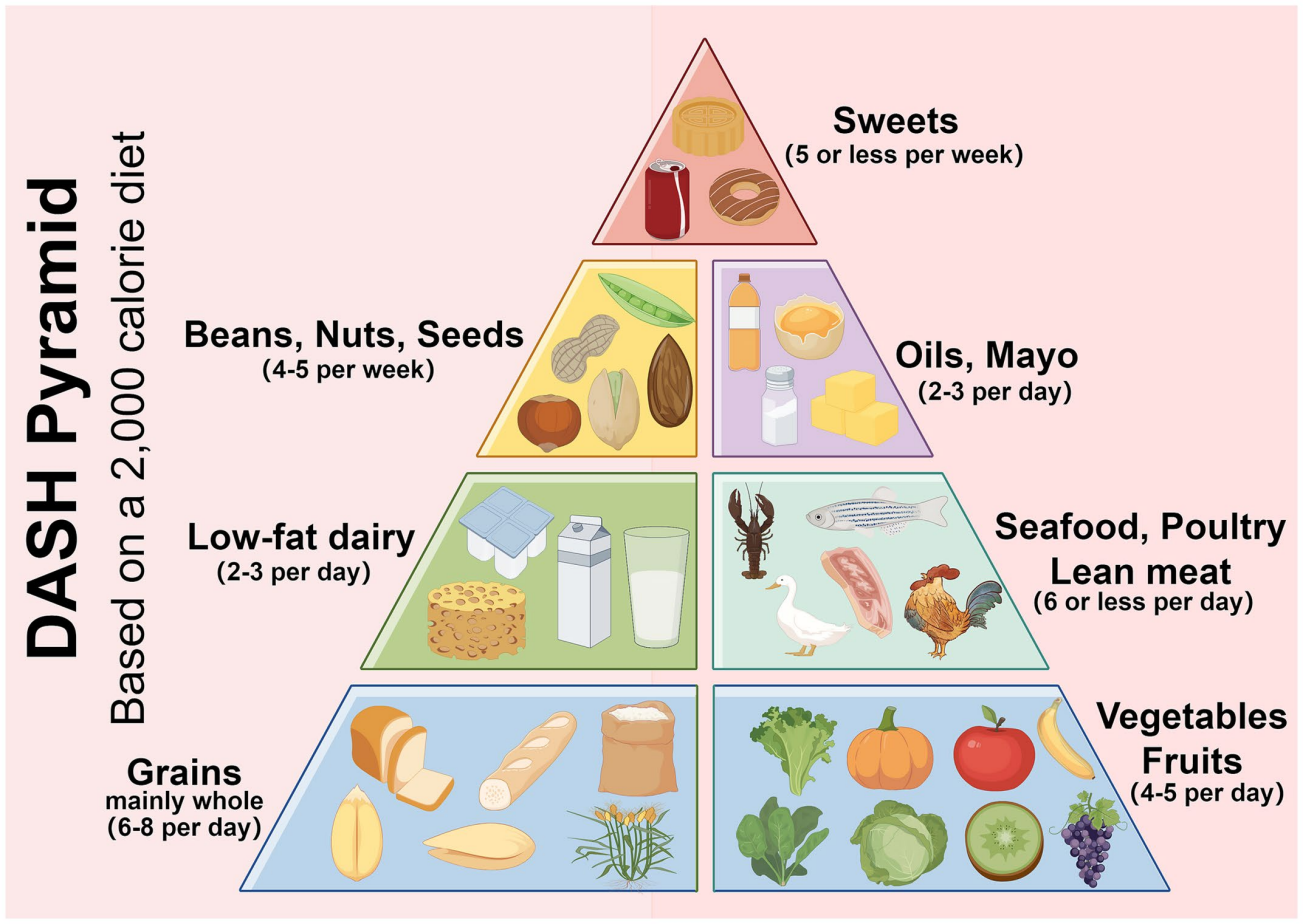
The DASH diet encourages eating more vegetables, fruits, legumes and low-fat dairy products; replacing refined white staples with whole grains; reducing red meat in favor of fish and chicken; cooking with vegetable oil; and limiting salt intake and sweets. Created with Figdraw.com.

The DASH diet is a dietary model with the goal of reducing blood pressure. Its core principles stress the consumption of foods rich in dietary fiber, potassium, calcium and magnesium, while limiting the intake of sodium, saturated fats and refined sugars. The DASH diet encourages increased consumption of fruits, vegetables, whole grains, low-fat dairy products, legumes, nuts, and lean meats and reduced consumption of red meat, processed foods, sugar-sweetened beverages, and foods high in salt (Figure 1) (8). The DASH diet was originally designed for hypertension, this antihypertensive effect was attributed mainly to sodium antagonism from high potassium foods and improved insulin sensitivity from dietary fiber, but its beneficial effects on metabolic syndrome, diabetes, and obesity have made it an important nonpharmacologic intervention for comprehensive metabolic management (9–14). The extended value of the DASH diet in the field of metabolic regulation and diabetes has gradually become a research hotspot. Future research is needed to further understand the molecular mechanisms, optimize the application strategies of the DASH diet, and explore the long-term benefits of the DASH diet in the prevention, treatment, and management of diabetes complications. Research in this area not only provides new ideas for the prevention and treatment of diabetes complications, but also lays the foundation for the integration of metabolic management strategies across diseases (Table 1).

**Table 1:** Basic information of included studies

**Study**	**Year**	**Country**	**Study design**	**Patients analysed (n)**	**Duration of years**	**Relative research** **results**	**Intervention** **effect**
Kenmoue et al.[59]	2022	Cameroon	Case– control	160	8 weeks	Decreases in BMI, SBP, DBP, LDL and TC 0.07 lower LDL/HDL ratio and 0.2 lower HbA1c levels Inversely associated with FPG, positively associated with insulin sensitivity Inverse association with metabolic syndrome, blood pressure and waist circumference Decreased TG, TC, VLDL, FFA The reduction of SBP and DBP. DASH+TRE group improved blood pressure diurnal rhythm DASH group experienced a significant reduction in the prevalence of the MetS and HBP Levels of pro-BNP, cardiac troponin I decreased A reduction in FPG, insulin, HOMA-IR, TG, TC, LDL DASH diet adherence have lower odds of DN A significant reduction in FLI, WC, weight, BMI, DBP, SBP, TG, TC and LDL Showed change in SBP and DBP. Decreased in AST, APRI and LAP Decreased in weight, BMI, TG, VLDL. Increased concentrations of TAC and GSH Decreased in weight, BMI, ALT, insulin levels, HOMA-IR. Increased in QUICKI. Decreased in TG, CRP, MDA. Increased in NO, GSH Decreased in TC, TG, LDL, LDL/HDL. Increased in HDL Lost more weight, consumed more Vitamin C, potassium, dietary vegetables	Effective Effective Effective Effective Effective Effective Effective Effective Effective Effective Effective Effective Effective Effective Effective Effective
Liese et al.[29]	2001-2005	United States	Cross- sectional study	2130	4		
Ramesh et al.[14]	N/A	United States	Longitudinal Evaluation Study	295	N/A		
Drehmer et al.[17]	2008-2010	Brasil	Cohort study	15105	2		
Hashemi et al.[22]	2016	Iran	Prospective study	80	12 weeks		
Zhou et al.[18]	2023	China	Prospective study	74	6 weeks		
Saneei et al.[60]	2020	Iran	Cross-over study	60	6 weeks		
Belanger et al.[50]	2014	United States	Prospective study	412	12 weeks		
Hosseinpour-Niazi et al.[27]	2021	Iran	Prospective study	300	16 weeks		
Mirzababaei et al.[13]	N/A	Iran	Case– control	210	N/A		
Sangouni et al.[30]	2022	Iran	Prospective study	60	4 weeks		
Badali et al.[25]	2019	Iran	Prospective study	62	8 weeks		
Asemi et al.[24]	2012	Iran	Prospective study	48	8 weeks		
Razavi et al.[49]	2014-2015	Iran	Prospective study	60	1 year		
Panbehkar-Jouybari et al.[48]	2016-2020	Iran	Cross- sectional study	4740	4 years		
Shenoy et al.[21]	2020	United States	Prospective study	81	12 weeks		

BMI, Body Mass Index; ALT, alanine aminotransaminase; HbA1c, glycosylated hemoglobin; SBP, systolic blood pressure; DBP, diastolic blood pressure; FPG, fasting plasma glucose; VLC, very low carbohydrate diet; DASH, dietary approaches to stop hypertension; HF-DASH, higher-fat, lower-carbohydrate modification of the DASH diet; Apo A, apolipoprotein A; pro-BNP, precursor of the brain natriuretic peptide; LDL, low-density lipoprotein; TG, triglycerides; TC, total cholesterol; HDL, high-density lipoprotein; VLDL, very low-density lipoprotein; FFA, free fatty acids; TRE: time-restricted eating; MetS, metabolic syndrome; HBP, high blood pressure; HOMA-IR, homoeostasis model of assessment-estimated insulin resistance; DN, diabetic nephropathy; FLI, fatty liver index; WC, waist circumference; AST, aspartate aminotransferase; APRI, platelet ratio index; LAP, lipid accumulation product; TAC, plasma total antioxidant capacity; GSH, glutathione; QUICKI, quantitative insulin sensitivity check index; CRP, C-reactive protein; MDA, malondialdehyde; NO, nitric oxide.

## 2 Clinical and preclinical evidence of the efficacy of DASH

### 2.1 The DASH diet reduces DN risk

Several studies have supported the association between DASH diet and reducing the risk of kidney disease in women with type 2 diabetes. One longitudinal study of women with diabetes found that the incidence of DN was 84% lower in the high-adherence DASH group than in the control group (13). This protective effect was independent of age, baseline renal function, and glycemic control level, suggesting a direct renal protective effect of the DASH diet (13, 14). The DASH diet significantly reduced urinary albumin excretion rate (UAER) and serum creatinine levels and improved estimated glomerular filtration rate (eGFR), indicating that it delayed renal decline (15, 16). Features of the DASH diet that are important in the diabetic women population, especially low-fat dairy products, provide sufficient calcium and vitamin D to help prevent osteoporosis while reducing the burden of renal calcium excretion (17). People with type 2 diabetes are often deficient in vitamin D, and supplementation is best done with activated vitamin D3 (e.g., Calcitriol). Several studies have shown that the DASH diet may indirectly reduce the risk of nephropathy in women with diabetes by improving metabolic and cardiovascular health indicators. In the future, further randomized controlled trials are needed to clarify the specific contributions of plant and non-plant components of DASH diet to DN, and to explore the effects of intervention at different stages of diabetes (13). Recent evidence, although not conclusive, suggests that potassium from natural sources is not harmful, even for chronic kidney disease (CKD). Its limitations can have paradoxical negative effects (14).

### 2.2 Blood pressure control

Several clinical trials have shown that the DASH diet has a obvious effect on reducing systolic blood pressure (SBP) and diastolic blood pressure (DBP). A randomized controlled trial in overweight and obese patients showed that subjects on the DASH diet alone had a reduction in SBP and DBP after the intervention, while those combined with time-restricted eating (DASH+ TRE group) had a greater reduction in blood pressure. The SBP and DBP decreased by 8.459 ± 4.260 mmHg and 9.459 ± 4.375 mmHg. The DASH+TRE group also improved circadian rhythm in blood pressure, suggesting that dietary interventions combined with meal timing adjustments may have a synergistic effect on blood pressure control (18). For women with gestational diabetes mellitus and a higher risk of pregnancy induced hypertension, the high-fiber DASH diet reduced the risk of pregnancy induced hypertension by 55% (8). Although the blood pressure lowering effect of the DASH diet has varied across studies, which possibly related to the intervention period, adherence, or population characteristics, increasing the consumption of fruits, vegetables, whole grains, and low-fat dairy products and reducing saturated fats and sodium have been widely established as effective strategies to improve blood pressure and metabolic health. The DASH diet lowers blood pressure through multiple mechanisms, such as regulating sodium-potassium balance, improving endothelial function, and reducing inflammation. As a result, substantial clinical evidence supports its use as a first-line non-pharmacological intervention for hypertension management (9, 19).

Elevated intake of fruits and vegetables can replace foods rich in salt and fats, reduce total calorie intake, and help control weight and lower blood pressure, these effects may be related to the vasodilatory and anti-inflammatory effects of bioactive components (nitrate and polyphenols) of fruits and vegetables (9, 16, 20, 21). The intake of fruits and vegetables in the DASH diet further regulates blood pressure levels by increasing dietary fiber and reducing sodium retention. Low-fat dairy products provide high-quality protein and calcium, and its protective effects may be achieved by regulating blood pressure and improving calcium metabolism. Calcium and vitamin D found in dairy products not only supplement bone mineral density but may also reduce hypertension risk through modulation of the renin-angiotensin system (RAS) (9, 19). Several intervention studies in overweight, obese and hypertensive patients have shown obvious blood pressure lowering effects of the DASH diet. The combined health benefits of DASH diet make it a priority for hypertension management.

### 2.3 Improvements in blood lipid and glucose levels

Macrovascular disease is a common complication in diabetic patients. However, the basis of macrovascular disease is due to lipid metabolism disorders. The DASH diet reduced the levels of triglyceride (TG), total cholesterol (TC), very low density lipoprotein (VLDL), and free fatty acid (FFA) after 12 weeks of intervention (*p* < 0.05), and the reduction of FFA was more significant in the DASH group (22). A meta-analysis of populations at high cardiovascular risk found that a low calorie diet (LCD) was associated with a statistically reduction in TG levels (23). A randomized controlled trial in overweight/obese women found that TG levels were clearly lower in the DASH diet group than in the control group (−10.0 mg/dL vs. +19.2 mg/dL, *p* < 0.001) (24). This effect may be related to the high dietary fiber, antioxidants and low saturated fat intake in the DASH diet, thereby improving lipid metabolism and reducing hepatic fat synthesis (24, 25). For TC regulation, the DASH diet reduces cholesterol absorption and promotes cholesterol excretion by increasing dietary fiber and phytosterol intake. The low saturated fat and high fiber components recommended by the DASH diet may reduce VLDL levels by inhibiting hepatic VLDL synthesis and promoting TG clearance in peripheral tissues. Nuts are rich in unsaturated fatty acids and plant protein, and their intake not only helps to improve lipid metabolism, but magnesium and dietary fiber in nuts may improve glucose uptake in muscle and the liver through activation of the AMPK pathway, thereby reducing the risk of T2DM, regulating insulin secretion, and improving insulin sensitivity (1, 13, 26). By adjusting the dietary structure, the DASH diet not only helps control blood pressure, but also effectively improves dyslipidemia, in particular reducing TG, TC and potentially VLDL levels, thereby reducing the risk of cardiovascular disease in diabetic patients.

The DASH diet can enhance the activity of insulin signaling pathway and reduce insulin resistance, thereby reducing HbA1c level. Multiple randomized controlled trials have shown that dietary fiber improves insulin sensitivity and reduces postprandial glucose fluctuations by delaying gastric emptying and regulating gut microbiota. The DASH diet can observably reduce fasting plasma glucose (FPG), insulin levels and homeostasis model assessment of insulin resistance (HOMA-IR), and improve quantitative insulin sensitivity check index (QUICKI), especially in people with obesity or hypertension complicated with type 2 diabetes (9). A 12 week intervention in overweight/obese patients with diabetes showed reductions in fasting plasma glucose (FPG), insulin injection dose, and HOMA-IR, and compared with the DASH diet, the legum-based DASH diet reduced FPG and HOMA-IR more significantly (27). The DASH diet with caloric restriction was found to result in reductions in body weight, which further reduced visceral fat accumulation and thereby indirectly enhanced insulin sensitivity (24). In a study of overweight or obese adolescents, strong adherence to the DASH diet reduced the risk of metabolically unhealthy obesity (MUO) by 91–92%, which was associated with markedly improved HOMA-IR and FPG levels (28).

The DASH diet stands out for long-term glycemic control. In patients with diabetes, 12-week DASH diet intervention resulted in a reduction in HbA1c, which occurred simultaneously with the improvement in the low-density lipoprotein/high-density lipoprotein (LDL/HDL) ratio, suggesting that blood glucose homeostasis is achieved through comprehensive metabolic regulation (29, 30). The glucose-lowering effects of the DASH diet varied between populations and intervention periods. Meta-analysis showed that the DASH diet reduced HbA1c by about 0.3% on average, but improved FPG more clearly (−5.5 mg/dL) (31). This may be related to the more stringent sodium restriction and higher proportion of whole grains consumed in the DASH diet (28). In women with gestational diabetes mellitus, the DASH diet can effectively reduce the risk of gestational hyperglycemia and postpartum type 2 diabetes mellitus by reducing HbA1c and regulating lipid profiles (8). The DASH diet may maintain a more stable HbA1c level by limiting intake of refined sugars and carbohydrates to reduce postprandial glucose fluctuations, especially in people at high risk of developing type 2 diabetes (32, 33). The DASH diet is an effective strategy for long-term glycemic and lipid control.

### 2.4 Arterial stiffness and vascular function

The low-sodium DASH diet may improve the arterial elasticity index, and the mechanism may be achieved through multiple pathways. Sodium intake is commonly recommended to be controlled to between 1,500 and 2,300 mg/d to regulate fluid balance and vascular tone (8, 9, 34). The reduction in blood pressure helps to reduce mechanical stress on the arterial walls, thereby improving vascular endothelial function and arterial elasticity (18, 23). This dietary pattern emphasizes the consumption of foods rich in potassium, magnesium, and calcium, which may enhance arterial compliance by regulating vascular smooth muscle tone and inhibiting oxidative stress (9). Polyphenols in fruits and vegetables, such as flavonoids, may reduce vascular injury by inhibiting oxidative stress and inflammation. The DASH diet combined with the TRE study was found to indirectly reduce arterial stiffness by reducing visceral fat accumulation and systemic inflammatory response (18, 20). The improvement of DASH diet on insulin sensitivity and metabolic syndrome may further promote the recovery of vascular endothelial function, thereby improving arterial elasticity (23). It was found that levels of short-chain acylcarnitines increased after intervention, and these metabolites were strongly associated with improved vasoconstricting function and arterial compliance (35). A systematic review showed that following the DASH dietary pattern with a 20% reduction in cardiovascular disease (CVD) risk, and the risk of coronary heart disease was reduced by 21%, which may be associated with improvement of metabolic syndrome and insulin sensitivity, reduction of inflammation. The low-sodium DASH diet reduces the risk of atherosclerosis by directly lowering blood pressure and optimizing ventricular-arterial coupling, regulates lipid profile, enhances antioxidant capacity to protect endothelial function, and metabolites such as acylcarnitine improve arterial elasticity through a variety of pathways.

### 2.5 Neuropathy and sleep health

Patients with diabetes mellitus often have neuropathy and sleep disorders. The association between the DASH diet and diabetic peripheral neuropathy was reflected in a reduction in the risk of diabetic neuropathy, including peripheral neuropathy, among women with type 2 diabetes who closely followed the DASH diet (36). Diabetic peripheral neuropathy (DPN) is the most common microvascular complication of diabetes. Its occurrence or development is related to long-term hyperglycemia, insulin resistance and systemic inflammation. Animal experiments have shown that DASH diet may improve neuroinflammation induced by high-fat diet, which is one of the important mechanisms of DPN (37). The DASH diets and may provide potential benefits for sleep disorders and neurodegenerative diseases through multiple mechanisms. This dietary pattern emphasizes the consumption of foods rich in antioxidants (such as polyphenols and vitamins C/E) or anti-inflammatory components (such as Omega-3 fatty acids) (26). Dietary polyunsaturated fatty acids such as Omega-3 and plant polyphenols may enhance neuroprotection by modulating synaptic plasticity, maintaining the integrity of neuronal outer membranes, and modulating insulin signaling (38, 39). DASH diet can improve blood pressure control in patients with metabolic syndrome, and hypertension is closely related to sleep apnea and neurodegenerative diseases (40). Animal studies have shown that high-fat diets disrupt peripheral circadian clock rhythms, and the diet’s low saturated fat and high fiber content may help maintain metabolic homeostasis, thereby reducing sleep cycle disruptions caused by metabolic abnormalities (41, 42). The DASH diet has a systemic anti-inflammatory effect, and intestinal inflammation has been associated with sleep disturbances and cognitive decline (43). The DASH diet may provide several intervention strategy for sleep disorders and neurodegenerative diseases by improving metabolism, anti-inflammation, anti-oxidation, and vascular protection.

### 2.6 Gestational diabetes mellitus

The DASH diet has been shown to have a effect on reducing fasting glucose during pregnancy. Studies have shown that the DASH diet improves metabolism and function during pregnancy through a variety of mechanisms, including regulating blood glucose, reducing inflammatory responses, and improving vascular function. In addition, adherence to the DASH diet was signally associated with a reduced risk of gestational diabetes mellitus (GDM), and the mechanism may involve reducing oxidative stress, increasing glutathione peroxidase activity and inhibiting chronic inflammation (reducing C-reactive protein levels) (8, 23). The DASH diet emphasizes balanced nutrition and avoids extreme restrictions, which is suitable for the nutritional needs of GDM. Low-fat dairy products and nuts provide sufficient calcium and healthy fats for fetal development (28, 44). The DASH diet also controls gestational weight gain and further reduces the risk of metabolic disorders (41). The DASH diet also reduced the risk of macrosomia (birth weight average loss of 109.5 g) during pregnancy, which may be related to the mechanism of action of regulating maternal blood glucose and inflammatory state. While available evidence supports metabolic benefits during pregnancy, specific implementation needs to be evaluated in the context of individual nutritional status (8, 45). Care should also be taken to adjust sodium intake to avoid the risk of edema during pregnancy (46).

### 2.7 Polycystic ovary syndrome

The positive effects of the DASH diet on reproductive health outcomes are multifaceted. There is a strong correlation between polycystic ovary syndrome (PCOS) and T2DM. Studies have found that about 50% of PCOS patients have insulin resistance, of which 30–40% have T2DM, which may be due to a combination of high insulin levels and excessive androgen levels caused by PCOS, resulting in insulin resistance and thus a strong link to the development of diabetes. Studies of PCOS patients have shown that the DASH diet can alleviate PCOS-related symptoms such as menstrual disturbances and infertility by improving insulin resistance and metabolic abnormalities. The DASH diet promotes ovulation recovery in PCOS patients by reducing androgen levels and improving insulin sensitivity. A trial of 48 women with PCOS showed that those on the DASH diet lost 4.4 kg of body weight and had a 15% reduction in androgen levels (24). The DASH diet increased plasma total antioxidant capacity (TAC) and glutathione (GSH) and reduced oxidative damage associated with PCOS (24). High fiber intake promotes the production of short chain fatty acids (SCFA), enhances intestinal barrier function and reduces endotoxemia, which indirectly improves metabolic and reproductive hormone balance (43, 44). These evidences suggest that the DASH diet, as a comprehensive dietary intervention strategy, has a synergistic effect on improving reproductive health problems by modulating metabolic pathways and inflammatory responses.

### 2.8 Diabetic retinopathy

Diabetic Retinopathy (DR) is a common microvascular complication of diabetes mellitus. Its occurrence and development are closely related to oxidative stress, inflammatory response and vascular endothelial injury induced by chronic hyperglycemia. The role of dietary interventions in the management of DR has attracted considerable attention in recent years. The DASH diet has become a potential strategy to delay the progression of DR because of its multi-target metabolic regulation characteristics. The DASH diet has antioxidant and anti-inflammatory effects, reducing the level of reactive oxygen species (ROS) and reducing oxidative stress damage to retinal microvessels. Its anti-inflammatory properties help to inhibit the expression of DR related inflammatory factors (such as IL-6 and TNF-α) to improve vascular endothelial function (1). The ingredients of the DASH diet have antiangiogenic effects, they may delay the pathological neovascularization in the late stage of DR by inhibiting the function of vascular endothelial growth factor (VEGF) (1). The DASH diet combined with nutraceuticals with anti-inflammatory and antiangiogenic properties (such as curcumin and resveratrol) can further strengthen the antioxidant defense system and reduce the levels of endothelial damage markers (such as VCAM-1 and ICAM-1) (1). Therefore, the DASH diet provides a scientific basis for the prevention and adjuvant treatment of DR by regulating metabolic disorders, oxidative stress, and inhibiting microvascular dysfunction.

## 3 Comprehensive analysis of the mechanism of action of the DASH diet

### 3.1 Synergistic effects of the nutrient components

**Figure 2:**
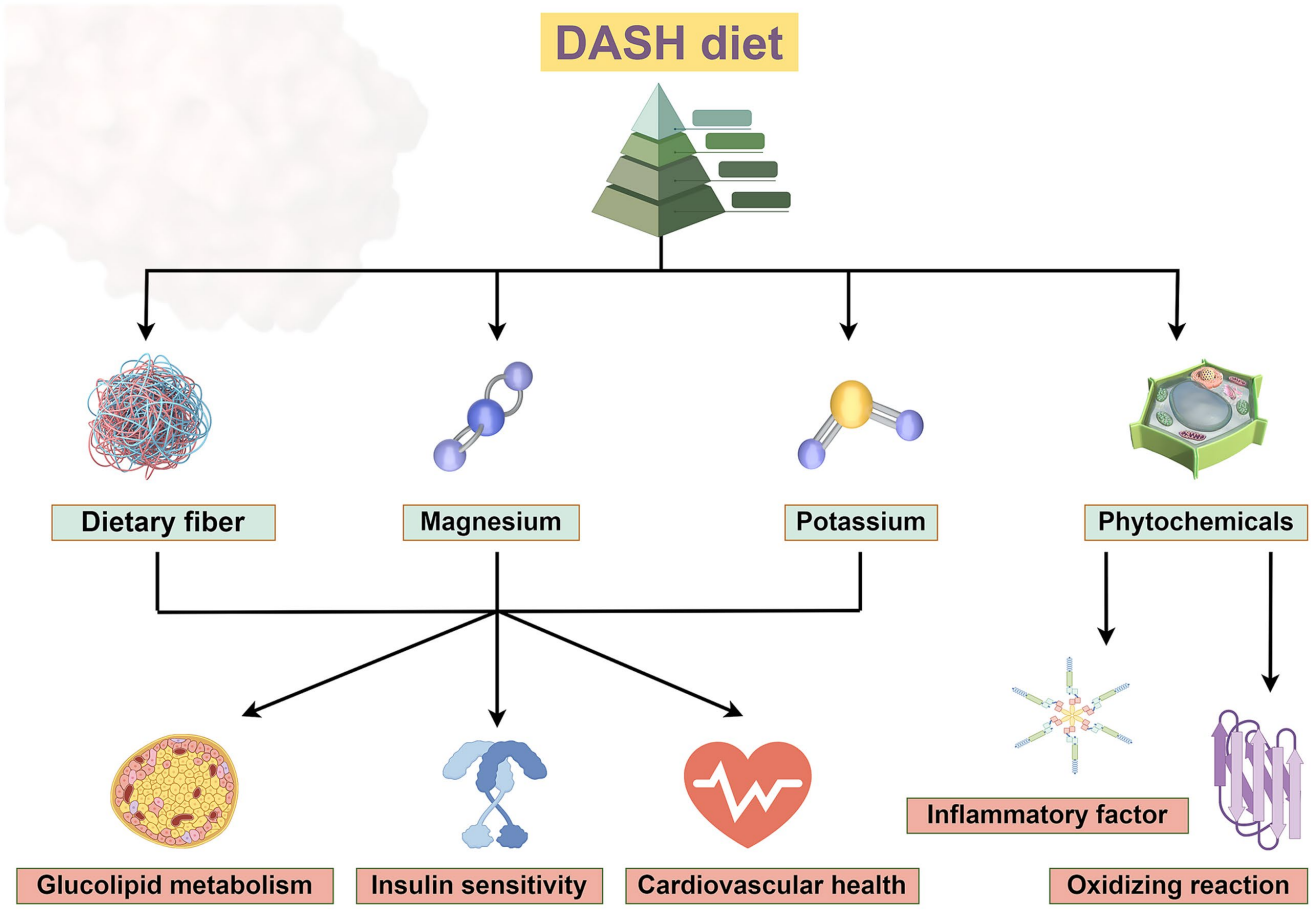
The DASH diet, which incorporates foods rich in dietary fiber, potassium and magnesium, improves glucose and lipid metabolism, insulin sensitivity and has beneficial effects on cardiovascular health. Phytochemicals can reduce the release of inflammatory factors and exert antioxidant effects. Created with Figdraw.com.

The metabolic regulation effect of DASH diet was mainly reflected in the comprehensive improvement of glucose and lipid metabolism, insulin sensitivity, and cardiovascular health (Figure 2). The DASH diet is rich in whole grains, vegetables, fruits, and legumes and provides high levels of soluble and insoluble dietary fiber. Foods such as nuts and whole grains in the DASH diet can enhance β-cell function and improve glucose homeostasis. Magnesium can act as a cofactor for more than 300 enzymes involved in glycolysis and insulin signaling, it is involved in glucose transport and insulin signaling pathways (13). Clinical trials have shown that FPG and HOMA-IR decreased in the DASH diet group, which may be related to the improvement of β-cell function by magnesium (27, 29). The synergistic effect of high dietary fiber, potassium, and magnesium may enhance the overall improved effect of the DASH diet on metabolic syndrome. The synergistic effect of these nutrients in the DASH diet may be more effective than supplementation alone. The combined intake of magnesium and potassium optimizes electrolyte balance, while dietary fiber promotes mineral absorption, creating a virtuous cycle of metabolic regulation. As a result, the DASH diet modulates metabolic disorders through the synergistic effect of elevated dietary fiber, potassium and magnesium. In conclusion, vegetables, fruits, nuts, and low-fat dairy products exert key metabolic protective effects in the DASH diet through multiple synergistic mechanisms, and are great valuable in improving glucose homeostasis, lipid metabolism, and cardiovascular health (9, 13, 17).

### 3.2 Protective mechanisms of the DASH diet

The DASH diet plays a protective role in metabolic diseases by significantly modulating oxidative stress and inflammatory markers through food components rich in antioxidant and anti-inflammatory properties. Antioxidant substances (e.g., lutein, carotenoids) in green leafy vegetables (e.g., spinach, kale) may improve insulin sensitivity and glycemic control by reducing oxidative stress (1, 22). Nitrates in beetroot have vasodilatory effects and indirectly reduce the risk of hypertension and cardiovascular disease (13, 17). The DASH diet provides an abundance of natural antioxidant components (e.g., polyphenols, vitamin C, vitamin E, and carotenoids) through a variety of plant based foods that neutralize free radicals and reduce oxidative stress levels, which also can reduce reactive oxygen species (ROS) production and inhibit NADPH-mediated lipid peroxidation in adipose tissue, thereby blocking the activation of inflammatory signaling pathways (1, 43). DASH diet-related metabolites (e.g., short-chain fatty acids, specific acylcarnitines) can reduce the vicious cycle of oxidative stress and inflammation by regulating mitochondrial function and insulin signaling pathway. Phytochemicals in the DASH diet may clearly inhibit inflammation and improve insulin sensitivity by modulating the gut microbiome and promoting short-chain fatty acid production (44, 45). The DASH diet has emerged as an effective dietary intervention strategy to improve metabolic diseases and chronic inflammatory states through the multi-component and multi-targeted antioxidant and anti-inflammatory effects. This evidence points to the central role of the DASH diet in the prevention and treatment of diabetes and the complications or comorbidities. The DASH diet also has shown potential benefits in reducing inflammatory factors and improving metabolic indicators related to coagulation function. Phytochemicals (such as polyphenols) enriched in the DASH diet exert anti-inflammatory and antioxidant effects through multiple mechanisms (Figure 2). Polyphenols ameliorate chronic inflammatory states by inhibiting proinflammatory signaling pathways such as nuclear factor kappa B (NF-κB), reducing the release of inflammatory factors, and reducing the levels of oxidative stress markers such as malondialdehyde. Polyphenols in plant (e.g., quercetin, anthocyanins) can activate the nuclear factor erythroid 2-related factor 2 (Nrf2) pathway and enhance the activity of endogenous antioxidant enzymes (e.g., superoxide dismutase and glutathione peroxidase) (44, 45). It effectively cleans up free radicals and inhibits the activity of proinflammatory transcription factors. Polyphenols in fruits and vegetables in the DASH diet can reduce inflammatory markers such as plasma CRP and TNF-α, and reduce systemic low-grade inflammation (46–50). DASH diet can also regulate the PI3K/Akt signaling pathway and TGF-β/Smad signaling pathway, which are involved in the progression of diabetic nephropathy and cardiomyopathy by promoting fibrosis and inflammation, and play a key role in various complications such as diabetic cardiomyopathy and nephropathy (51–53). These pathways can also regulate insulin sensitivity, cell survival and metabolic homeostasis. The DASH diet alleviates diabetic microvascular complications and improves inflammation and oxidative stress by inhibiting the interaction between advanced glycation end products (AGE) and its receptor (RAGE) (51, 54, 55). In addition, DASH diet also involves HIF-1 signaling pathway, which may play a part in diabetic wound healing disorders and ischemic complications (51, 56). In the Adipocytokine signaling pathway, DASH diet can regulate adipokines (such as leptin and adiponectin), and regulate insulin resistance and metabolic disorders through this pathway. The DASH diet may also ameliorate inflammation, fibrosis, metabolic disorders, and vascular damage in diabetic complications through genes such as VEGFA, FOXO1, and TP53 (51, 55, 57, 58). Together, these mechanisms act on complications such as cardiomyopathy, nephropathy, retinopathy, and neuropathy.

## 4 Challenges to the special population

The low-sodium DASH diet shows potential feasibility in improving blood pressure and glucose control in elderly patients with diabetes. Low-sodium adjustment (sodium intake ≤1,500 mg/d) can further optimize the effect of blood pressure control, and may be beneficial for renal protection in elderly diabetic patients with CKD, while hypertension and CKD are common complications or comorbidities in elderly patients with diabetes (20, 59). In elderly patients, the DASH diet markedly reduced FPG, HOMA-IR and HbA1c, which may delay the progression of diabetes complications (34). DASH diet can reduce TC, LDL and TG and increase HDL, which is highly beneficial for elderly patients with diabetes mellitus and metabolic syndrome (48, 60, 61). Older patients may need to adjust the texture of foods (e.g., easy to chew vegetables, mush nuts) because of dental problems or swallowing difficulties, and ensure protein intake to prevent amyotrophy (24, 28). In frail older adults with diabetes, healthy fats such as olive oil can be increased appropriately to maintain energy intake (43). The DASH diet combined with aerobic exercise or anaerobic exercise can enhance weight loss, improve insulin sensitivity and cardiopulmonary function. However, low-intensity activities (e.g., walking and water exercise) should be tailored to the physical capacity of older people with diabetes (20). The low-sodium DASH diet can reduce the dose of antihypertensive and glucose-lowering medications, but close monitoring is needed to avoid the risk of hypotension or hypoglycemia (59). But long-term compliance may be affected by dietary habits, chewing function, or socioeconomic factors in the elderly. Nutrition guidance, simplified dietary programs, and family support are needed to improve the feasibility (62). Overall, it needs to be combined with clinical evaluation and regular monitoring to optimize the efficacy.

The latest research suggests a less restricted intake of sodium and water in chronic heart failure (CHF) patients. The DASH diet itself recommends low sodium (≤2,300 mg/d), but it should be further adjusted according to the patient’s heart function classification. Studies have shown that the sodium-restricted DASH diet can improve cardiovascular function, reduce blood pressure and cardiac afterload in patients with hypertension (35). CHF patients recommended to choose foods with high nutrient density (such as fruit purees, lean meat) rather than low-fat dairy products to reduce fluid burden. The DASH diet, which is rich in potassium (e.g., bananas, spinach) and magnesium (e.g., nuts, whole grains), improves myocardial electrophysiological stability but requires monitoring of serum potassium levels (especially in those using potassium-sparing diuretics or those with renal insufficiency). For lactose intolerance, choosing lactose-free, low-fat dairy products (e.g., hard cheese, Greek yogurt) or calcium/vitamin D-fortified plant alternatives (e.g., almond milk, soy milk) can meet DASH dietary calcium and protein requirements while avoiding lactose-induced gastrointestinal symptoms (14). For severe intolerance, calcium and vitamin D3 supplementation with dark green vegetables (kale, broccoli) and fish (sardines, salmon) is recommended. High-quality protein can be supplemented with beans, fish, poultry, and eggs, avoiding reliance on dairy products. Serum potassium, body weight, and intestinal tolerance should be regularly monitored during supplementation, and individualized adjustments should be made in conjunction with a nutritionist when necessary (28). However, it should be noted that plant substitutes may lead to higher oxalate intake (26). It is recommended to adjust the dietary structure in combination with 24-h urine test. One meta-analysis showed that high adherence was associated with a 12% lower risk of stroke compared with low adherence, and another study showed that each 4-point increase in the DASH score on a 40-point scale was associated with a 4% lower risk of stroke (63). There was a linear inverse association between DASH dietary score and stroke risk, indicating a stronger protective effect with higher adherence. The effect of DASH diet on stroke risk reduction was better in Asian population than in Western population (63). Low adherence to the DASH diet is significantly associated with high levels of disability in patients with ischemic stroke, so adherence to the DASH diet may reduce the occurrence of disability (63, 64). The DASH diet can reduce the incidence of stroke mainly through the management of risk factors (such as blood pressure lowering and lipid lowering), and may improve the prognosis and quality of life of patients with stroke (65, 66). However, its role as an independent treatment method still needs more long-term clinical research to support (63, 64, 67).

There is limited evidence from direct studies on the DASH diet in different diabetes subtypes, such as LADA (latent autoimmune diabetes in adults) and MODY (maturity onset diabetes of the young), but the potential role can be speculated based on metabolic regulation mechanisms. The core features of the DASH diet show improvements in insulin sensitivity and β-cell function, which may be of particular value for metabolic control in patients with LADA because of both autoimmune destruction and insulin resistance features (16). LADA belongs to the class of autoimmune diabetes, but some patients may be accompanied by metabolic syndrome (46). The anti-inflammatory characteristics of the DASH diet may help alleviate chronic inflammatory states, and it is necessary to monitor indicators of autoimmune activity (13). The DASH diet may affect insulin secretion and glucose tolerance by regulating the acylcarnitine and fatty acid metabolic profile, which has reference significance for the diet management of MODY subtypes involving β-cell function gene mutations, such as HNF1A-MODY (68). Improvements in obesity-related metabolic abnormalities with the DASH diet have been demonstrated in adolescent populations, and may have synergistic therapeutic effects with specific diabetes subtypes associated with obesity, such as HNF1A-MODY (68). It should be noted that for subtypes of diabetes requiring tight carbohydrate control (e.g., GCK-MODY), these patients intake of fruits and the carbohydrate content of whole grains may need to be adjusted individually. MODY is caused by a single gene mutation and is mainly characterized by defective insulin secretion, but some subtypes (such as hepatocyte nuclear factor 1α mutation) may be accompanied by abnormal lipid metabolism and optimize carbohydrate source in combination with blood glucose fluctuation pattern. In addition, features of low sodium and high potassium may help alleviate the risk of hypertension due to renal tubular dysfunction in some MODY patients (59). However, MODY patients may have individualized nutritional requirements, so it is necessary to adjust the proportions of macronutrients. In the future, precision nutrition research should be conducted based on the pathophysiological characteristics of different subtypes of diabetes.

## 5 Recommendations for the DASH diet

### 5.1 Cultural background and dietary preferences

The cultural background and dietary preferences of the patients should be sufficiently considered to ensure long-term compliance and intervention effectiveness of the DASH diet. In terms of cultural adaptation, Asian cultures have replaced whole grains with brown rice, buckwheat, or millet, increased the consumption of soy products (e.g., tofu, natto) and local vegetables (e.g., cabbage, kale), and reduced the sodium content of pickled foods (69). Mediterranean cultures retain olive oil, fish and nuts as the main sources of fat, combined with local vegetables (e.g., tomatoes, aubergines) and whole-wheat pasta to increase intake of anti-inflammatory ingredients (70). The DASH diet explicitly targets the prevention and management of hypertension, with an emphasis on reducing blood pressure by limiting sodium intake and increasing nutrients such as potassium, calcium, magnesium, and fiber. In contrast, the Mediterranean diet focuses more on overall health maintenance, including the prevention of cardiovascular disease, obesity, and cognitive decline. Latin American cultures have replaced refined flour products with tortilla, increased the consumption of black beans, quinoa and tropical fruits (e.g., papaya, guava), reduced the use of processed meats (8). For the integration of patient preference and feasibility, for patients who prefer sweet taste, it is recommended to replace added sugar with natural fruits (such as berries and citrus), and for patients who prefer salty taste, vanilla, lemon juice, or low-sodium flavoring (such as turmeric and garlic) can be used to improve the flavor (23). For vegetarians, increase the intake of plant proteins (such as lentils and chickpeas) and nuts. For those who are lactose intolerant, choose plant milk (such as almond milk and soy milk) instead of low-fat dairy products (7, 17). Recommend local ingredients (e.g., sweet potatoes, green leafy vegetables) and prefabricated food (e.g., multigrain porridge, vegetable soups) to reduce decreased adherence due to cost or time constraints (71). The DASH diet requires caloric restriction to achieve weight loss goals, but long-term hypocaloric intake may induce hunger and lead to decreased adherence (49). Severe restriction of red meat and saturated fat may reduce the intake of nutrients such as iron and vitamin B12, and deficiency should be prevented with alternative foods or supplements (28, 48). Adolescents, pregnant women, or chronic disease patients who have followed the DASH diet for a long time need to adjust energy and nutrient ratios to avoid developmental or metabolic abnormalities (72). It recommends gradually increasing the proportion of fruits and vegetables and whole grains and reducing processed foods, and adapting the DASH diet framework to regional dietary characteristics. Regular monitoring of electrolyte, liver and kidney function and nutritional status should be carried out, nutrition education and community support should be combined to maintain compliance (73). It is crucial to emphasize that dietary recommendations are not one-size-fits-all. Individual factors such as age, sex, baseline health status, cultural background, and personal preferences can significantly influence the efficacy and adherence to any dietary pattern. Therefore, the translation of these findings into clinical practice should be guided by healthcare professionals, such as registered dietitians or nutritionists, to ensure personalized and sustainable dietary advice. Through dynamic assessment of patients’ food feasibility, cooking habits and health goals, a personalized nutritional intervention path with scientific and cultural adaptability can be constructed (49, 74). Encourage the whole family to participate in meal planning, such as group sourcing of healthy ingredients, creating weekly menus, and enhancing networking support (59, 60). Regular food diary analysis were used to adjust the protocol in time and solve difficulties in implementation.

### 5.2 DASH diet combined with exercise therapy

The study found that while the DASH diet reduced waist circumference, body fat percentage, and visceral fat, the combination of aerobic exercise and caloric restriction resulted in greater weight loss (75). This synergistic effect may result from the dual regulation of increased energy expenditure by exercise and decreased energy intake by dietary control (24, 76). DASH diet combined with physical activity can synergistically reduce the risk of T2DM by increasing energy expenditure and optimizing glucose regulation pathways (such as improving insulin secretion and sensitivity) (33). Long-term adherence to the DASH diet and exercise lifestyle can effectively reduce the risk of cardiovascular disease and chronic kidney disease by improving chronic low-grade inflammation and endothelial function (77, 78). Overall, the DASH diet combined with exercise can effectively intervene in metabolism, inflammation and cardiovascular function. It is better for health than diet or exercise interventions alone.

## 6 Conclusion

**Figure 3:**
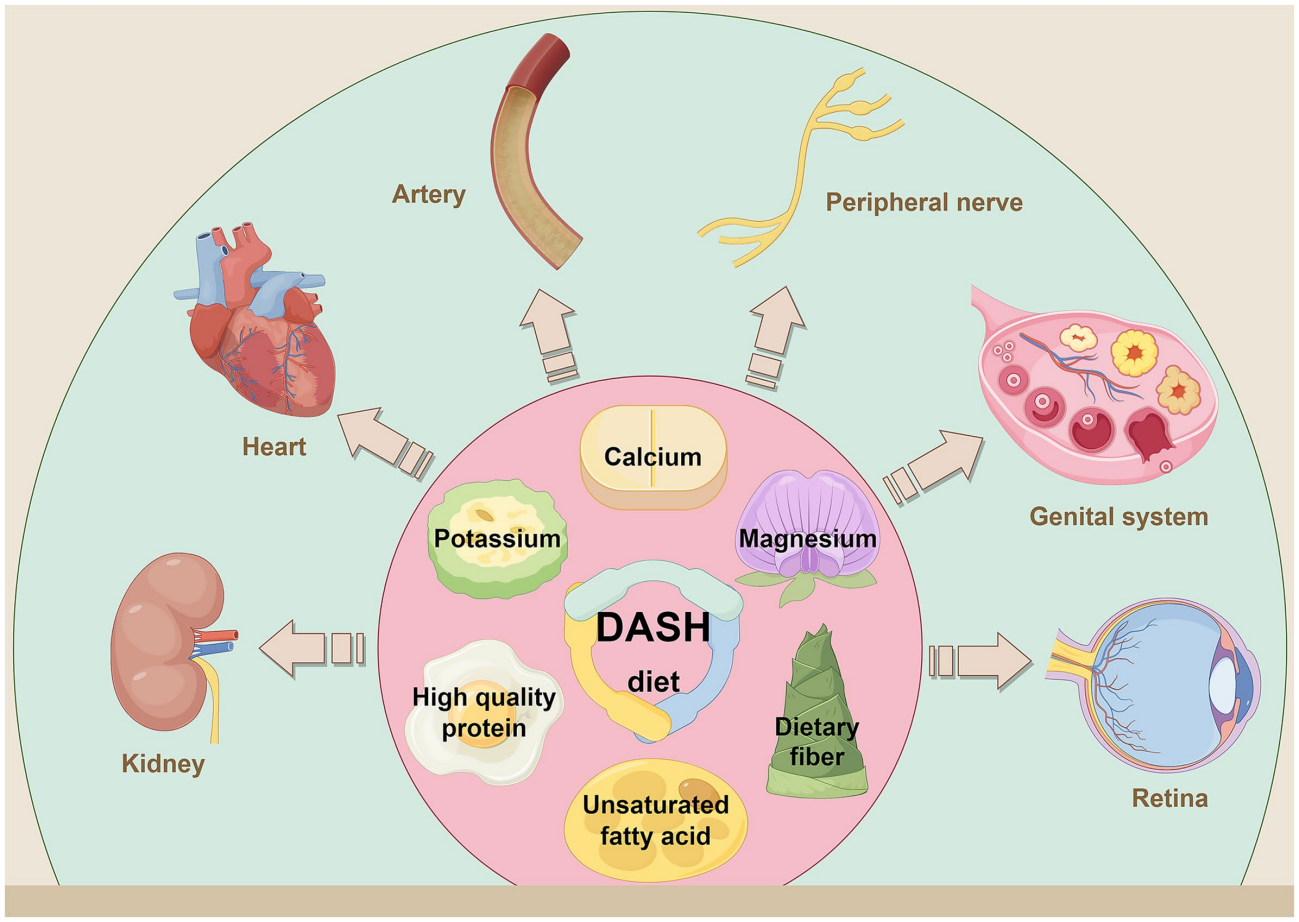
By consuming foods high in dietary fiber, potassium, calcium, magnesium, high quality protein and unsaturated fatty acids, while limiting sodium, saturated fat and refined sugar. The DASH diet reduces the risk of diabetes related complications and comorbidities through a multipathway mechanism. It has shown a clear protective effect in the prevention and treatment of nephropathy, cardiovascular diseases, arteriosclerosis, peripheral neuropathy, PCOS and retinopathy. Created with Figdraw.com.

**Figure 4:**
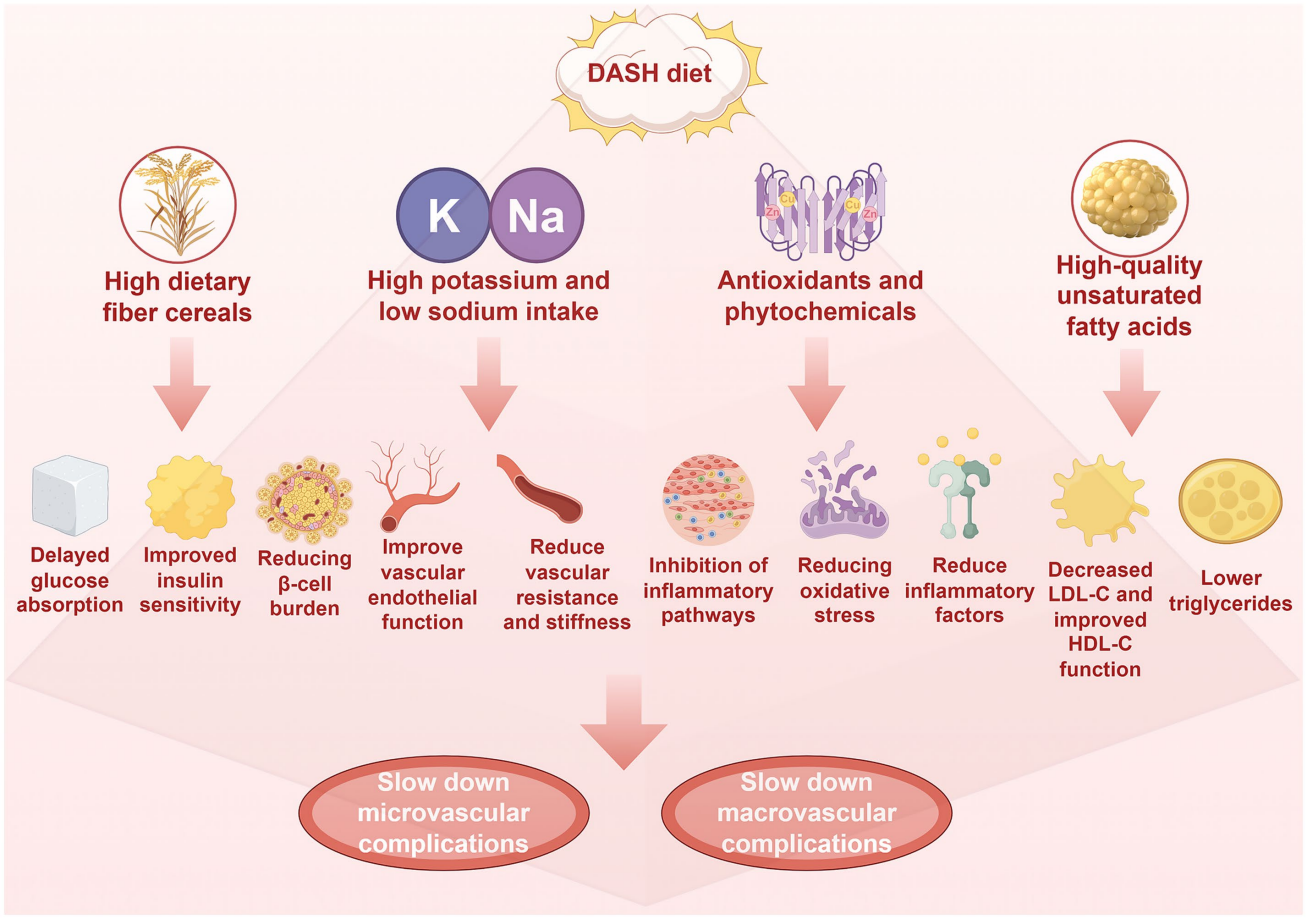
The DASH diet is characterized by high dietary fiber, potassium, magnesium and low sodium. High dietary fiber is beneficial to blood glucose control, which can delay glucose absorption, improve insulin sensitivity and reduce the burden of pancreatic β-cells, while high potassium, magnesium and low sodium are beneficial to blood pressure control and vascular function. Results in increased nitric oxide bioavailability, improved endothelial function, and decreased vascular resistance and stiffness. In addition, the DASH diet is rich in antioxidants, plant compounds and high-quality unsaturated fatty acids. These antioxidants and plant compounds can play anti-inflammatory and anti-oxidative effects, inhibit inflammatory pathways such as NF-κB, reduce oxidative stress and reduce inflammatory factors (such as TNF-α and IL-6). High-quality unsaturated fatty acids can reduce LDL-C and triglyceride, and increase HDL-C levels. Through the above mechanisms, it will eventually slow down the occurrence of microvascular and macrovascular complications of diabetes, which is more conducive to slow down the decline of glomerular filtration rate and urine protein, protect fundus microvessels and improve nerve function, delay the process of atherosclerosis, reduce the risk of coronary heart disease, reduce the risk of stroke, and improve peripheral artery disease. Created with Figdraw.com.

The DASH diet reduced the risk of diabetes related complications through multipathway mechanisms, especially in the prevention and treatment of kidney disease, cardiovascular events, and metabolic disorders (Figure 3). The protective effect of DASH diet may be due to the synergistic effect of the overall dietary structure, including minerals rich in potassium, magnesium, calcium, and antioxidant polyphenols, which together act on multiple pathophysiological processes. In conclusion, the safety, effectiveness and sustainability of the DASH diet make it an important dietary pattern for chronic disease management, and the DASH diet provides comprehensive protection against diabetes and diabetes complications or comorbidities (Figure 4).
